# Assessment of potentially preventable hospitalizations in the regional hospital of Saint-Louis, Senegal

**DOI:** 10.11604/pamj.2017.27.125.10360

**Published:** 2017-06-16

**Authors:** Abdoul Aziz Ndiaye, Mouhameth Bakhoum, Alioune Badara Tall, Ndeye Fatou Ngom-Gueye, Mohamed Sidy Seck, Boubacar Gueye, Awa Diop-BA, Awa Gaye, Gallo Papa Sow, Lamine Gueye, Anta Tal-Dia

**Affiliations:** 1 Department of Community Health, Alioune Diop University, Bambey, Senegal; 2 Faculty of Health Sciences, Gaston Berger University, Saint-Louis, Senegal; 3 Public Health Department, Cheikh Anta Diop University, Dakar, Senegal

**Keywords:** Potentially preventable hospitalizations, non-communicable diseases, Saint-Louis

## Abstract

**Introduction:**

The "potentially preventable hospitalizations (PPH)'' are hospital admissions that could have been avoided through effective primary care given at the appropriate time. Non-communicable diseases (NCDs), causes of PPH, are the leading cause of death worldwide with significant socioeconomic consequences especially in developing countries. This study aimed to assess the burden of potentially preventable hospitalizations in the St. Louis regional hospital.

**Methods:**

This was a descriptive cross-sectional study. The surveyed population consisted of all patients older than one year, admitted to St. Louis hospital for more than four (04) hours time between January 20 and April 30, 2015. Patients hospitalized in surgery (general surgery, ENT, ophthalmology), maternity and neonatology, as well as those who refused or were unable to participate in the study were excluded.

**Results:**

The study included one hundred forty four (144) individuals with an average age of 54.68±15 years (17-88 years) and sex ratio woman/man of 1.21. The PPH represented 54% of all hospitalizations. The main causes of hospitalizations were diabetes with 22.1%, chronic kidney disease 12%, hypertension 10.9%, Stroke 6.4% and finally broncho-pulmonary diseases 2.6%. The average length of stay was 6.68±5.51 days. The average distance between the residence and the hospital was 26.51±60KM with a median of 3.5KM. The average cost of care was Euros 104.583 ±83.51. For 61.10%, it was a first hospitalization and for 30.60%, a second one. The Knowledge about signs of disease severity had changed significantly at the end of hospitalization, from 29% at the beginning to 98% at the end of stay in hospital. As for the means of prevention, 30.55% reported knowing them before their hospitalization and 68% after hospitalization.

**Conclusion:**

Potentially preventable hospitalizations are a heavy burden for the population of St. Louis. Their negative social and economic impacts may hinder health policies initiated to relieve vulnerable groups. Their prevention should be a national priority.

## Introduction

The "potentially preventable hospitalizations" (PPH) are hospital admissions that could have been avoided through efficient primary care dispensed at the appropriate time [1–5]. The concept of conditions to preventable hospitalizations or (PHC = Preventable hospitalization conditions) appeared in 1990 in the United States; the idea was to use as an indicator of the quality of ambulatory care, hospitalization rate for these pathological conditions [2–5]. Since then, preventable hospitalization rate is used to measure the quality of outpatient care in many Western countries [6–10]. Most studies conducted since 1990 have been inspired by PHC conditions identified by Billings (Billings and al, 1990) or Weissman (Weissman and al, 1992) [2, 3, 6] allowing to extract them from hospital data and to characterize risk zones [1, 3–5, 11, 12]. The non-communicable diseases (NCDs), sources of potentially preventable hospitalizations are the leading cause of death worldwide [13]. According to the World Health Organization (WHO) report on the non-communicable diseases, they represent the leading cause of death, killing more people than all other causes combined each year [13]. Of the 56 million deaths that occurred worldwide in 2012, 68% were from non-communicable diseases (NCDs), mainly cardiovascular diseases, cancers, diabetes and chronic lung diseases [13]. NCDs have a disproportionate impact on low and middle-income countries, which identify nearly 80% of deaths from these diseases, that is 29 million. They are the leading causes of death in all regions except Africa [14]. However, according to current projections, this continent is expected to register a significant increase in the number of NCD deaths by 2020. This should exceed the cumulative number of deaths due to communicable diseases, nutritional diseases and maternal and perinatal mortality. By 2030, NCDs will become the most common cause of death [14]. These diseases have reached epidemic level proportions while they could be substantially reduced, and that millions of lives could be saved and huge suffering avoided, with a decrease in risk factors, early detection and timely treatment [6, 9, 15].

The important thing is not to count then all potentially preventable hospitalizations for all diseases, but to identify those diseases for which hospitalizations are translating well, a priori, of a deficit of ambulatory care [1, 3, 6]. Socially, these diseases are very costly for patients, their families and for the society [9, 15, 16]. In 2012, they were responsible for 38 million (68%) of deaths in the world. Over 40% of these deaths were premature, about 16 million, that is to say, they occurred before the age of 70. On deaths from non-communicable diseases worldwide, almost three quarters, about 28 million, as well as the majority of premature deaths (82%) occurred in low and middle-income countries [13]. In France the PPH represents 10.58% whereas in England it was estimated to 13.17% in 2006 and since then figures have not diminished [6, 15–18]. The consequences worldwide remain alarming, while in Senegal, the review of the literature fails to find information on the potentially preventable hospitalizations. By contrast a study has shown a higher frequency of high blood pressure (24.1%). Diabetes (9.7%) and chronic kidney disease (22.4%) and most of the screened patients were unaware of their condition [19, 20]. According to WHO, if nothing changes in countries with low and middle income, it is estimated that from 2011 to 2025, the cumulative economic losses due to non-communicable diseases will reach US $ 7,000 billion [13]. The huge cost of inaction is much higher than what it would cost each year for the implementation of a series of interventions to more effectively reduce the burden attributable to non-communicable diseases (US $ 11.2 billion per year) [13]. However, a strategy focused on promoting health could significantly reduce the burden of potentially preventable hospitalizations [6]. Various studies on potentially preventable hospitalizations were performed all over the world except in Africa. Thus, the main objective of this work was to assess the potentially preventable hospitalizations in the St. Louis regional hospital. The use of qualitative and quantitative approaches helped to better achieve this objective.

## Methods

This was a descriptive cross-sectional study on potentially preventable hospitalizations at Saint-Louis Regional Hospital. The target population consisted of all patients admitted to St. Louis hospital during the period from January 20^th^ to April 30^th^, 2015. Were included patients older than one year and admitted to the hospital for more than 4 hours time. The inpatient in surgery (general surgery, ENT, ophthalmology), maternity and neonatology as well as patients who had refused or were unable to answer were excluded from the study. The survey was comprehensive including all eligible patients during the study period. Initially, eligible patients were identified. Then, based on the diagnosis made by the physician, those admitted for potentially preventable diseases (PPH) were asked if they were able to answer; otherwise, the persons accompanying answered for them. The identification of eligible patients and those admitted for a condition related to a PPH has determined the frequency of PPHs and that of each condition. Diseases included in this study responsible for PPHs are diabetes, high blood pressure, stroke, chronic obstructive pulmonary disease and kidney disease. Diabetes was defined by fasting glucose ≥1.26 g / l or being under oral anti-diabetic agents or insulin therapy [21]. High blood pressure was defined by systolic blood pressure (SBP) ≥ 140 mm Hg and or diastolic blood pressure (DBP) ≥ 90 mm Hg, or being on antihypertensive therapy [21].

Chronic obstructive pulmonary disease (COPD) was defined by a non-infectious chronic cough associated with respiratory dyspnea. Chronic kidney disease was defined regardless of its cause, by the presence for more than 3 months, markers of kidney damage or decreased of glomerular filtration rate (GFR) below 60 ml /min/1.73m^2^[22, 23]. Stroke is a condition that occurs in the aftermath of an attack of brain arteries. Two types of stroke are distinguished: ischemic stroke is a motor deficit in the context of heart disease or hypertension; hemorrhagic stroke involves an alteration of consciousness in the context of heart disease or hypertension. A structured questionnaire was used to collect information on the socio-demographic characteristics of the patient, the reason for hospitalization, duration of stay, the average distance between the residence and the hospital, the cost of care. The knowledge of the severity of these pathologies targeted as well as means of prevention were explored. The participation was free and voluntary and always started by the signing of a letter of informed consent. Data collection went well, in most of the cases; interview was done with the patients themselves, for rare bedridden, accompanying persons were able to answer in their places. Data was entered using the sphinx software, and analyzed using the R software

## Results

During the study period, 267 eligible patients were admitted at the concerned departments of Saint-Louis regional hospital. The average age was 54.68 years with a standard deviation of 15 years, a median of 56 years. The first quartile was 46 years and the 3^rd^ quartile of 65 years. The 17-39 years accounted for 17.36% of patients; the 40-59 years for 42.36% and finally the 60-70 years for 40.28% out of the total. Just over 82% of participants had more than 39 years. The sex ratio female/male was 1.2; that is 54.9% women and 45.1% women. Among them 144 presented PPH. Table 1 shows the socio-demographic characteristics of participants interviewed. Of the 144 patients, 68.1% were from the Wolof ethnic group, 20.1% from the Alpulars and 6.9% from the Maures. The married represented 76.4% of patients, widowed 11.8%, single 7.6% and divorced 4.2%. Almost 30% were craftsmen or traders, 27.78% housewives and 22.2% of retired persons. More than half of patients (59%) were illiterate and a quarter had a primary school level. Just under two thirds of patients (63.9%) had a monthly income below 76.22 Euros; 21.5% between 76.22 and 152.45 Euros; 9.7% between 152.45 and 228.67 Euros, 4.9% and an equal or greater income of 228.67 Euros. Table 2 shows the frequency of potentially preventable hospitalizations in the pattern. The percentages were determined by the number of hospitalization during the study period (267). Considering all hospitalizations, the frequency of PPH was estimated at 54%, those related to diabetes 22.1%, chronic kidney disease 12% /, hypertension 10.9, stroke 6.4% and broncho-pneumonia 2.6%. The average length of stay of PPH was 6.68 ± 5.51 days, with a time of stay ranging from 1 to 35 days.

**Table 1:** Socio-demographic characteristics of the participants

Variable	Number	Percentage
**Age group**		
17 – 39 years	25	17.36%
40 – 59 years	61	42.36%
60 – 88 years	58	40.28%
**Gender**		
Male	65	45.10%
Woman	79	54.90%
**Ethnics**		
Wolof	98	68.10%
Alpular	29	20.10%
Maure	10	6.94%
Others	7	4.86%
**Marital Status**		
Married	110	76.40%
Widowed	17	11.80%
Single	11	7.60%
Divorced	6	4.20%
**Profession**		
Craftsmen/Traders	43	29.86%
Housewives	40	27.78%
Retired	32	22.22%
Others	29	20.14%
**Level of studies**		
Illiterate	85	59.00%
Primary School Studies	36	25.00%
Secondary School Studies	14	9.75%
Advanced Studies	9	6.25%

**Table 2:** Frequency of PPH according to the underlying disease

Diseases	Number of cases	Percentage
Diabetes	59	22.1%
Chronic kidney disease	32	12%
High Blood Pressure	29	10.9%
Stroke	17	6.4%
Broncho pneumonia	7	2.6%
Total	144	54%

**Figure 1:**
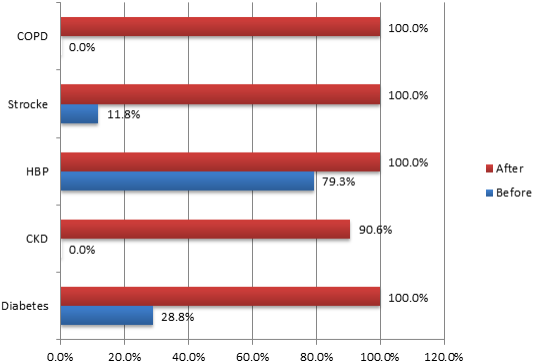
Distribution of knowledge level of severity before and after hospitalization

The median length of stay was 5.5 days; the 1^st^ quartile in two days and the third in 10 days. For 45.80% of patients, it was less than 5 days; between 5 and 10 days for 27.80%; between 10 and 15 days for 17.40% and finally 15 days and more for 9% of patients. The study of the frequency of hospitalizations for the same pathology showed that 61.10% of patients were admitted only once in the last 12 months, 30.60% twice and 8.30% three times or more. The average cost of care was 104.55 Euros with a standard deviation of 83.51; extreme values were 7.62 and 453.54 Euros. The median was 91.47 Euros, the first quartile to 35.06 and the 3^rd^quartile 160.07 Euros. Of the 144 patients, 36.80% spent less than 60.98 Euros for their care, 25% spent between 60.98 Euros and 121.96, and 18.8% between 121.96 and 182.94 Euros, 18.8% and 19.40% over 182.94Euros. Table 3 below shows the severity level of knowledge compared to their conditions before and during their hospital stay. Regarding the signs of severity, 102 of 144 patients, i.e. 70.8% were unaware of the signs of severity of their disease against 42 patients i.e. 29.2% before their hospitalization. The level of knowledge has increased during the stay from 29.20% to 97.90%. For diabetes, the level rose from 28.8% to 100%, chronic kidney disease from 0% to 90.6%, hypertension 79.3% to 100%, stroke of 11.76% 100% and finally broncho-pneumonia (COPD) 0% to 100%. The Figure 1 best illustrates the significant improvement of the level of knowledge of the severity of the conditions of avoidable hospitalizations at the end of stay in hospital. Regarding the means of prevention, 44 of 144 patients i.e. 30.55% were reported knowing them before their hospitalization and 98 patients i.e. 68% after their hospitalization. For diabetes, the level of knowledge of prevention methods increased from 37.28% to 89.47%, for HBP 68.97% to 100% and for stroke 11.76% to 29.40%. For patients with chronic kidney disease, the level increased from 0% to 37.50% and for the pulmonary disease (broncho-pneumonia) from 0% to 14.29%.

**Table 3:** Distribution of PPH depending on the cause and the knowledge level of severity before and after hospitalization

Cause of admission	Knowledge before	Knowledge after	number
Diabetes	17 (28.80%)	59 (100%)	59
Chronic kidney disease	0 (0.0%)	29 (90.60%)	32
High Blood pressure	23 (79.30%)	29 (100%)	29
Stroke	2 (11.76%)	17 (100%)	17
COPD	0 (0.0%)	7 (100%)	7
Total	42 (29.20%)	141 (97.90%)	144

## Discussion

The PPH is a heavy burden for the St. Louis hospital, in fact, 54% of internal medicine admissions were due to conditions that would have been managed in ambulatory care. This finding confirms trends in the epidemiology of non-communicable diseases in the area [24, 25]. Diabetes, which caused more PPH with 22.1%, is common in the population of St. Louis. Two studies conducted have shown prevalence between 10.4% and 12.7% [24, 25]. The WHO report in 2014 on NCDs, estimated prevalence of 10% with diabetes a risk of an increase in developing countries by 2025 [13, 19, 26, 27]. Chronic kidney disease was the second leading cause of PPH in St. Louis hospital (12%). A recent study showed a prevalence of 16% at regional level and 4.9% in the district of Saint Louis [24, 25]. The implementation of a hemodialysis center in the hospital could explain the high frequency of chronic kidney disease patients admitted in internal medicine. This chronic kidney disease represents one of major public health challenges of the 21^st^ century and is associated with significant morbidity and cardiovascular mortality [25, 27, 28].

High blood pressure (HBP) was the third cause of PPH (10.9%). This result correlates with the high prevalence observed in the population of Saint-Louis, ranging between 39.1% and 46% [24, 25]. It is responsible for significant morbidity in the world [13]. Modifiable risk factors such as excessive intake of salt, fat, obesity and sedentary lifestyle characterize this population, especially women. Strokes have led 6.4% of internal medicine hospitalizations. The survey of cardiovascular risk factors in the city of St. Louis in 2010 reported a prevalence of 3.2% [24]. This difference could be explained by the geographic origin of the patients, in fact, a significant proportion is coming from rural areas. Globally, 6.7 million strokes were recorded in 2014. Broncho-pneumonia has resulted in 2.6% of hospitalizations. More than 3 million people died of chronic obstructive pulmonary disease (COPD) in 2012, corresponding to 6% of all deaths in the world this year and over 90% of COPD deaths occur in countries with low and middle income [14].

## Conclusion

The PPH is a heavy burden for the population of Saint-Louis, also for the health system. The significant increase in knowledge of severity and means of prevention of pathologies responsible for such hospitalization suggests that the establishment of a community based program centered on health promotion, and quality of basic health cares could greatly reduce the social and health and economic impact of the PPH.

### What is known about this topic

- The PPH have a strong correlation with NCDs constitute a global emergency, especially in developing countries;
- Their situation and the risk areas are described especially in the North.

### What this study adds

- This was an exploratory study in a national hospital and the results have revealed strong recommendations on access prevention and health promotion;
- Other hospitals have initiated the same investigation.

## Competing interests

The authors declare no competing interests.
